# *Sargassum horneri* C. Agardh space capacity estimation reveals that thallus surface area varies with wet weight

**DOI:** 10.1371/journal.pone.0199103

**Published:** 2018-06-19

**Authors:** Min Xu, Shuji Sasa, Teruhisa Komatsu

**Affiliations:** 1 CAS Key Laboratory of Marine Ecology and Environmental Sciences, Institute of Oceanology, Chinese Academy of Sciences, Qingdao, P.R. China; 2 Laboratory for Marine Ecology and Environmental Science, Qingdao National Laboratory for Marine Science and Technology, Qingdao, P.R. China; 3 Atmosphere and Ocean Research Institute, The University of Tokyo, Chiba, Japan; 4 Hebei Provincial Research Institute for Engineering Technology of Coastal Ecology Rehabilitation, Tangshan, P.R. China; University of Waikato, NEW ZEALAND

## Abstract

*Sargassum horneri* C. Agardh is an important commercial edible seaweed species in east Asia. Benthic beds and floating rafts in coastal areas make excellent habitats for marine organisms to feed, hide, and spawn. Many commercially important fish species such as Japanese anchovy (*Engraulis japonicus*), yellowtail (*Seriola quinqueradiata*), and Japanese horse mackerel (*Trachurus japonicus*) live in seaweed beds. Chinese and Japanese fisherman rely on *S*. *horneri* beds as productive fish harvest areas. The Zhejiang government in China set a total allowable catch standard, to preserve the Ma’an Islands ecosystem, which is a marine protected area. In this study we analysed the association between weight and one-sided surface area of *S*. *horneri* beds, and calculated the ratio of one-sided surface area to change in wet weight over time. We collected samples from December 2014 to May 2015. Approximately 1 g of *S*. *horneri* biomass provided ~15 cm^2^ of one-sided surface area available to marine organisms. These calculations can be used as a reference regarding potential space to improve total allowable catch standard management in *S*. *horneri* beds, through the estimation of space capacity of seaweed beds.

## Introduction

*Sargassum* seaweed beds are important coastal habitats for a variety of marine organisms; in these habitats marine organisms feed, hide, and spawn [1–6]. Among *Sargassum* species, *Sargassum horneri* (Turner) C. Agardh is a seaweed species (Class: *Phaeophyceae*; Order: *Fucales*; Family: *Sargassaceae*) with an annual cycle that belongs to the order *Fucales* [7]. *S*. *horneri* is widely distributed along the northeast coasts of Asia (from China to Russia), the Korean Peninsula, and Japan (except Taiwan and the Ryukyu Archipelago)[8]. *S*. *horneri* has aggressively spread throughout southern California (USA), and Baja California, (Mexico) since 2003. *S*. *horneri* poses a major threat to the sustainability of native marine ecosystems in this region [9–10].

*S*. *horneri* beds adjust water temperature [11–13], pH [14], dissolved oxygen content [15], downward illumination [15], and water flow [13] of the surroundings. *S*. *horneri* beds form an underwater forest with 3–7m thalli, above hard substrate, *S*. *horneri* lush thalli serve as a natural habitat for many sessile organisms, such as caprellids, and gammarids [16], which attract juvenile fish to feed.

Local fishermen inhabiting the Nanji Islands, Zhejiang Province, China, call *S*. *horneri* the ‘DingXiang House’; ‘DingXiang’ is a Chinese word describing that Japanese anchovy (*Engraulis japonicus*) juveniles prefer to school in *S*. *horneri* beds.

Floating seaweed rafts provide an important ocean habitat for a diversity of fish [17–19]. In spring and early summer, most of the floating seaweed rafts on the Japanese coast contain detached *S*. *horneri* thalli [20–21]. Floating seaweed rafts in the east China Sea contain only *S*. *horneri* thalli [22–24,8] onto which commercially important pelagic fish lay their eggs. These fish species include flying fish, Pacific saury (*Cololabis saira*), and Japanese halfbeak (*Hyporhamphus sajori*); these fish species spawn eggs that contain filaments that attach them to the floating seaweed rafts [23]. Yellowtail (*Seriola quinqueradiata*) and Japanese horse mackerel (*Trachurus japonicus*) juveniles use these floating seaweed rafts as habitat [17]. Japanese fishermen rely on floating *S*. *horneri* rafts to collect and rear juvenile fish of these commercially important species into adulthood.

Moreover, in the Tohoku Region of Honshu Island (Japan), local people maintain the tradition to process *S*. *horneri* rafts for food [25]. Since 1185 A.D., in the Kashiwazaki area of the Niigata prefecture (Japan), local people have collected beached *S*. *horneri* rafts as seasonal food in winter [26].

The wet biomass of mature *S*. *horneri* is a potential industrial material for extracting fucoidans and alginic acid, which are widely used as chemicals added to foods, medicines and organic fertilizers[27]. Due to all of its potential benefits, it is of interest to exploit and properly manage this coastal resource of Chinese and Japanese fisheries in a sustainable way.

The Chinese government has listed the Ma’an Islands, Zhejiang Province, China, as a marine protected area to better manage *S*. *horneri* beds; it has asked local fishermen to make fishing notes. All fishing activities in *S*. *horneri* beds must follow the total allowable catch standard principle [27–31]. However, there is a lack of detailed information to implement a proper management strategy using a sustainable controlled catch. It is important to note that seaweed of longer thalli (~1–3 m) and increased wet weight (~200–1000 g) would represent an increase in surface area for sessile organisms to attach. This study estimated the surface area of thalli of varying wet weights of samples collected from December 2014 to May 2015. These biological data are important to sustainably manage these seaweed beds as an important fisheries resource.

## Materials and methods

### Study site and sampling

The field survey was approved by Shimoda Marine Research Center, Tsukuba University.

The study site (34°39'58" N, 138°56'31" E) was located in Shidagaura Cove, Shimoda, Izu Peninsula, Japan. The study site contains a rocky shore sheltered from waves, close to the Shimoda Marine Research Centre at Tsukuba University. The work details were described in [32]. Field surveys were conducted at intervals of *ca*. 1 month from December 15^th^, 2014, through May 28^th^, 2015. During each survey, scuba divers randomly sampled *ca*. 20–40 *S*. *horneri*.

Thalli collected by the divers were numbered individually and transported to a laboratory where the weight of each of them was determined. Samples were kept at −30°C until analysis. Samples were left in the air for thawing. We used a scale (CR-5000WP, Custom) to determine wet weights of the thalli (±2 g) after absorbing surface water with a paper towel.

One-sided surface area of the thalli was measured using ImageJ 6.4 software (National Institutes of Health, Bethesda, MD, USA; http://imagej.nih.gov/ij) by converting the pixels into the area. Digital photographs of each thallus, accompanied by a 30 cm ruler for scale calibration, were taken. One-sided surface area was measured using a calibration equation obtained from the square of a known area (5x5cm). We prepared five photographs of each individual that were connected horizontally using image processing software (Photoshop CS; Adobe Systems Inc., San Jose, CA, USA). The mean area of the five images was used as the one-sided surface area of a thallus.

### Statistical analysis

We used one-way ANOVA to compare the monthly ratios. Pearson’s correlation coefficient analysis was applied to determine the relationships between one-sided surface area and weight. A *p* value < 0.05 was considered significant. We used one-way analysis of variance (post-hoc test) to detect differences in the one-sided surface area as a function of weight ratio, which was calculated independently in monthly samplings.

## Results and discussion

**Fig 1:**
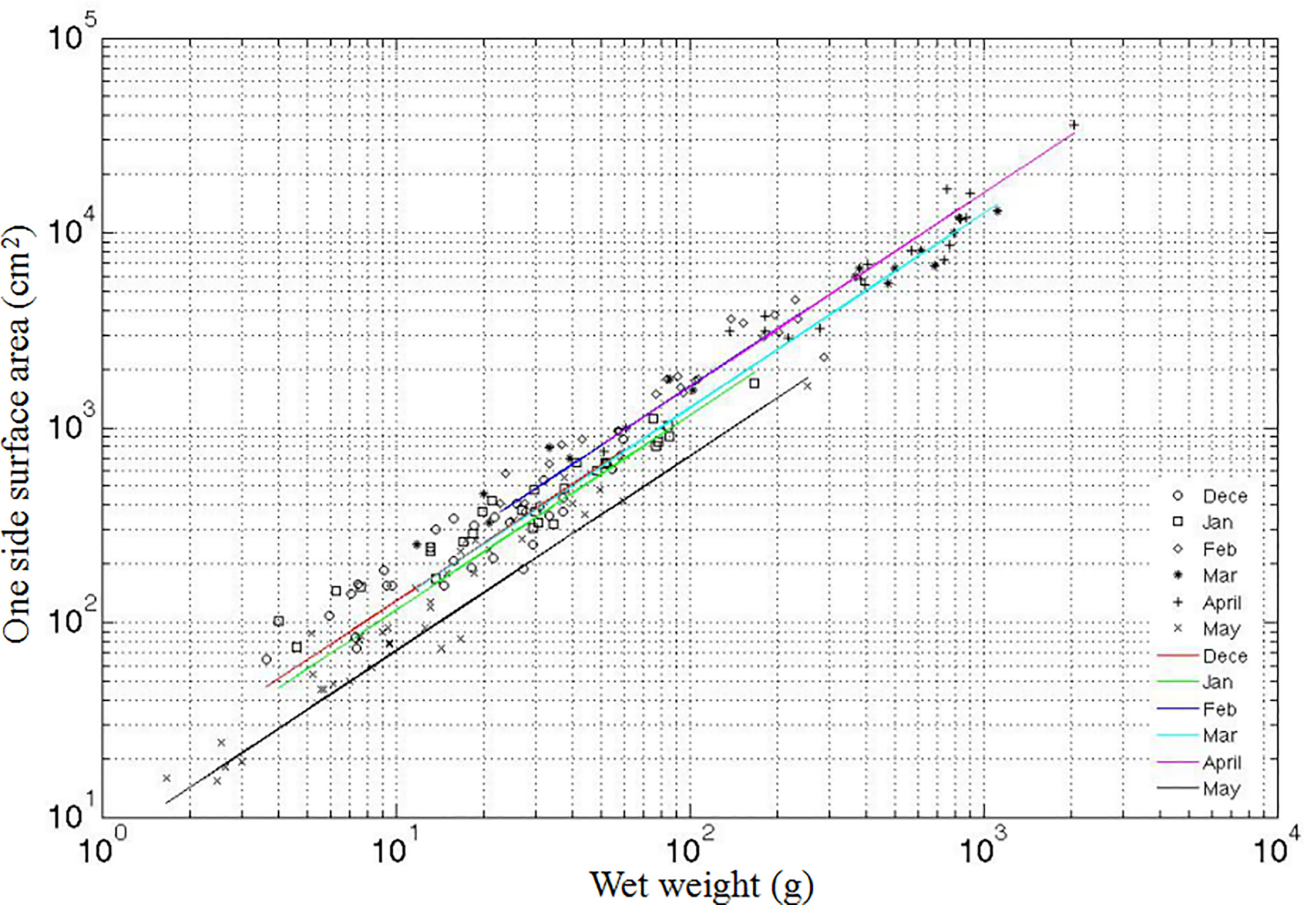
Log plot of wet weight (W) (g) versus one-sided surface area (S) (m^2^) from December 2014 to May 2015. Solid lines are regression fits. Correlations in December 2014 (*S* = 12.818 × *W*, r^2^ = 0.91, *p* < 0.001), January 2015 (*S* = 11.543 × *W*, r^2^ = 0.98, *p* < 0.001), February 2015 (*S* = 16.215 × *W*, r^2^ = 0.67, *p* < 0.001), March 2015 (*S* = 12.62 × *W*, r^2^ = 0.64, *p* < 0.001), April 2015 (*S* = 16.05 × *W*, r^2^ = 0.96, *p* < 0.001) and May 2015 (*S* = 7.1355 × *W*, r^2^ = 0.97, *p* < 0.001).

One-sided surface area increased as wet weight increased. One-sided surface area was linearly related to wet weight (Fig 1). The ratios of one-sided surface area to wet weight were significantly different and varied independently between months (*p < 0*.*001*).

**Fig 2:**
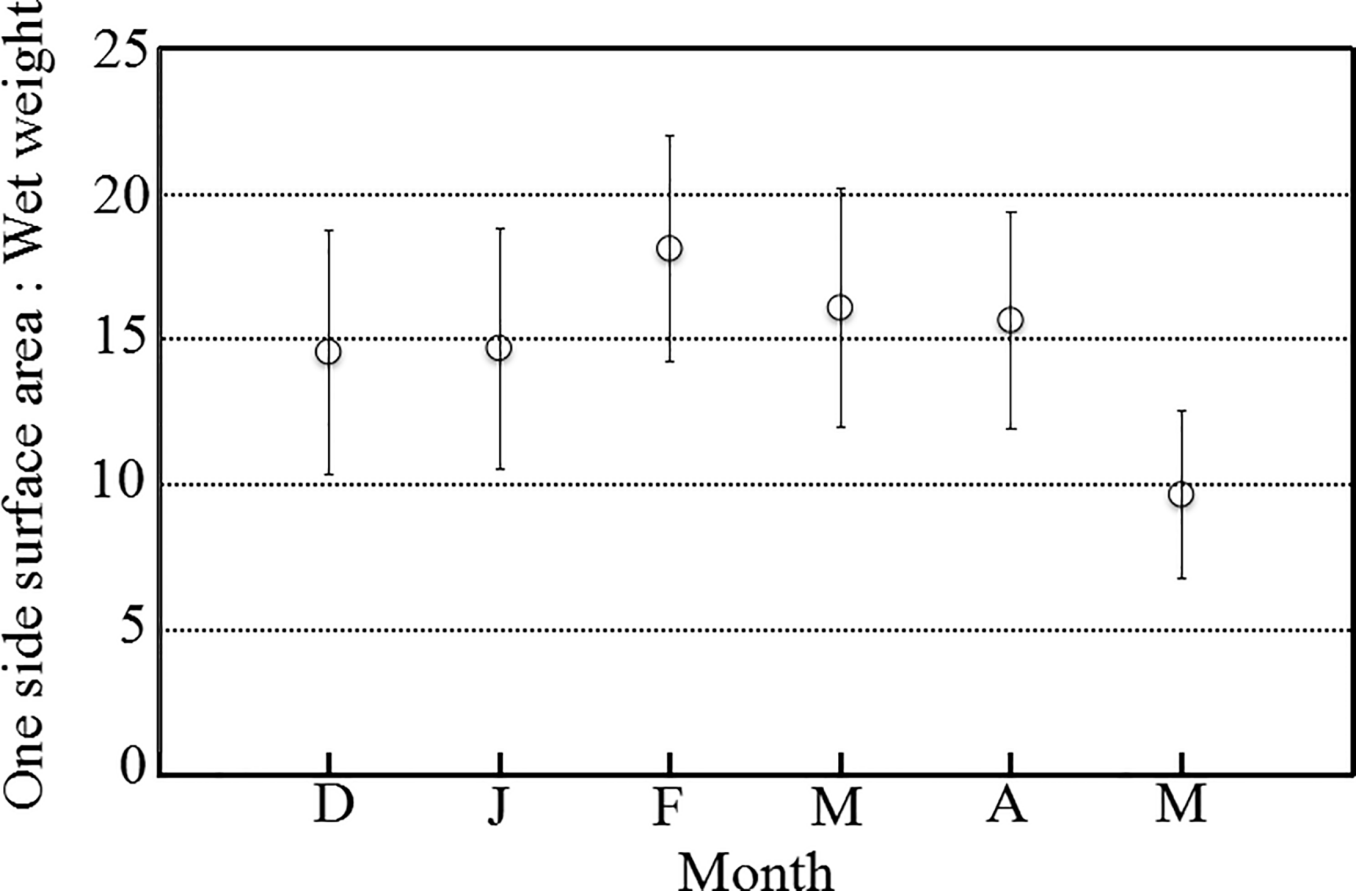
Monthly variations in the ratio (cm^2^ g^−1^) of one-sided surface area (cm^2^) to wet weight (g) from December 2014 to May 2015. Values are means ± standard deviations.

The mean ratios of one-sided surface area to wet weight were near 15 cm^2^ g^-1^ (F = 10.31, p < 0.001), and the monthly standard deviations were similar (Fig 2). Most branches of thalli were lost in May. In May only the holdfast and some decaying branches were attached to the substrate. Therefore the ratio of one-sided surface area to wet weight was lowest during May.

*S*. *horneri* beds are regarded as seabed forests in subtidal areas, and floating *S*. *horneri* rafts in the ocean are good habitats for a diversity of fish. Marine bacteria and diatoms attach to the surface of seaweed [33]. The numbers of phytoplankton and diatoms attached to the surface of *S*. *horneri* fronds have been estimated to into 10^2–3^ and 1.4–4.8 × 10^3^ per g, respectively [34]. Many of the macrosessile fauna feed on the covering film of bacteria, diatoms, and protozoa [35]. Many phytal amphipods, such as *Caprella decipiens*, *Caprella danilevskii*, *Caprella tsugarensis*, *Ampithoe orientalis*, *Aoroides columbiae*, and *Pontogeneia rostrata*, attach to the mid-part of the thallus; their numbers are linearly related to *S*. *horneri* biomass [16]. Lush foliage and a complex branch structure provide small refuges for many sessile organisms to escape from predators [36]. Thus, *S*. *horneri* beds are micro-ecosystems that attract juvenile fish to school. The *S*. *horneri* thallus biomass bloomed in late spring to early summer, increasing from ~20 g in December to ~600 g in April in this study; thus, the surface area of the fronds became available for marine organisms.

Wet weight was linearly related to one-sided surface area during several months. In our study the one-sided surface area to wet weight ratio was estimated at ~15 cm^2^ g^−1^ during the growing season (roughly from December to May) [37]. This value was higher than that reported for the seaweeds *Chondrus crispus* Stackhouse (~7 cm^2^ g^−1^) and *Mastocarpus stellatus* (Stack. In With) Guiry (~3 cm^2^ g^−1^) [38], which shows that *S*. *horneri* has a larger plate-attaching surface.

*S*. *horneri* is an annual seaweed species. Its large biomass provides ~6 months (December to May) of habitat for fish to feed, spawn, and avoid predators. Many Ma’an Island fishermen, particularly older fishermen with less physical ability, rely on *S*. *horneri* beds to catch juvenile fish to earn a living. These older Chinese fishermen are precluded from traditional social welfare, such as medical and retirement income, due to their farmer status. Thus, fishing in *S*. *horneri* beds is a part-time source of income and an important food source. It is very important to preserve and manage these beds and the marine organisms living in the beds sustainably.

The Zhejiang government set a total allowance catch standard to manage the Ma’an Island seaweed beds. However, there is a lack of good scientific information on how many fish should be captured from these beds and how many juveniles should be released into the seaweed beds to replenish the fish communities. Space and food are the most fundamental needs of marine organisms, particularly fish. The surface area of an *S*. *horneri* thallus frond not only provides space for other organisms, but also supports the food needs of invertebrates living in the thalli and the fish living in the seaweed beds. The economically important species *Sebastes schlegelii* and *Hexagrammos otakii* were prey on gastropods such as *Gammaridea* and *Caprellidae* living in the leaves of the seaweed [39–40] in Ma’an islands. It will be necessary to improve total allowance catch management on the *S*. *horneri* seaweed beds in a sustainable way.

## Supporting information

S1 File Dataset. Included all the data used in the paper.
(XLSX)
